# Recent developments in time-of-flight PET

**DOI:** 10.1186/s40658-016-0138-3

**Published:** 2016-02-16

**Authors:** S. Vandenberghe, E. Mikhaylova, E. D’Hoe, P. Mollet, J. S. Karp

**Affiliations:** ELIS-IMINDS-Medical IT-IBITECH Ghent University, De Pintelaan 185, Blok B, Gent, 9000 Belgium; Department of Radiology, University of Pennsylvania, Philadelphia, Pennsylvania USA

**Keywords:** PET, Time-of-flight, Reconstruction

## Abstract

While the first time-of-flight (TOF)-positron emission tomography (PET) systems were already built in the early 1980s, limited clinical studies were acquired on these scanners. PET was still a research tool, and the available TOF-PET systems were experimental. Due to a combination of low stopping power and limited spatial resolution (caused by limited light output of the scintillators), these systems could not compete with bismuth germanate (BGO)-based PET scanners. Developments on TOF system were limited for about a decade but started again around 2000. The combination of fast photomultipliers, scintillators with high density, modern electronics, and faster computing power for image reconstruction have made it possible to introduce this principle in clinical TOF-PET systems. This paper reviews recent developments in system design, image reconstruction, corrections, and the potential in new applications for TOF-PET. After explaining the basic principles of time-of-flight, the difficulties in detector technology and electronics to obtain a good and stable timing resolution are shortly explained. The available clinical systems and prototypes under development are described in detail. The development of this type of PET scanner also requires modified image reconstruction with accurate modeling and correction methods. The additional dimension introduced by the time difference motivates a shift from sinogram- to listmode-based reconstruction. This reconstruction is however rather slow and therefore rebinning techniques specific for TOF data have been proposed. The main motivation for TOF-PET remains the large potential for image quality improvement and more accurate quantification for a given number of counts. The gain is related to the ratio of object size and spatial extent of the TOF kernel and is therefore particularly relevant for heavy patients, where image quality degrades significantly due to increased attenuation (low counts) and high scatter fractions. The original calculations for the gain were based on analytical methods. Recent publications for iterative reconstruction have shown that it is difficult to quantify TOF gain into one factor. The gain depends on the measured distribution, the location within the object, and the count rate. In a clinical situation, the gain can be used to either increase the standardized uptake value (SUV) or reduce the image acquisition time or administered dose. The localized nature of the TOF kernel makes it possible to utilize local tomography reconstruction or to separate emission from transmission data. The introduction of TOF also improves the joint estimation of transmission and emission images from emission data only. TOF is also interesting for new applications of PET-like isotopes with low branching ratio for positron fraction. The local nature also reduces the need for fine angular sampling, which makes TOF interesting for limited angle situations like breast PET and online dose imaging in proton or hadron therapy. The aim of this review is to introduce the reader in an educational way into the topic of TOF-PET and to give an overview of the benefits and new opportunities in using this additional information.

## Introduction

**Fig. 1:**
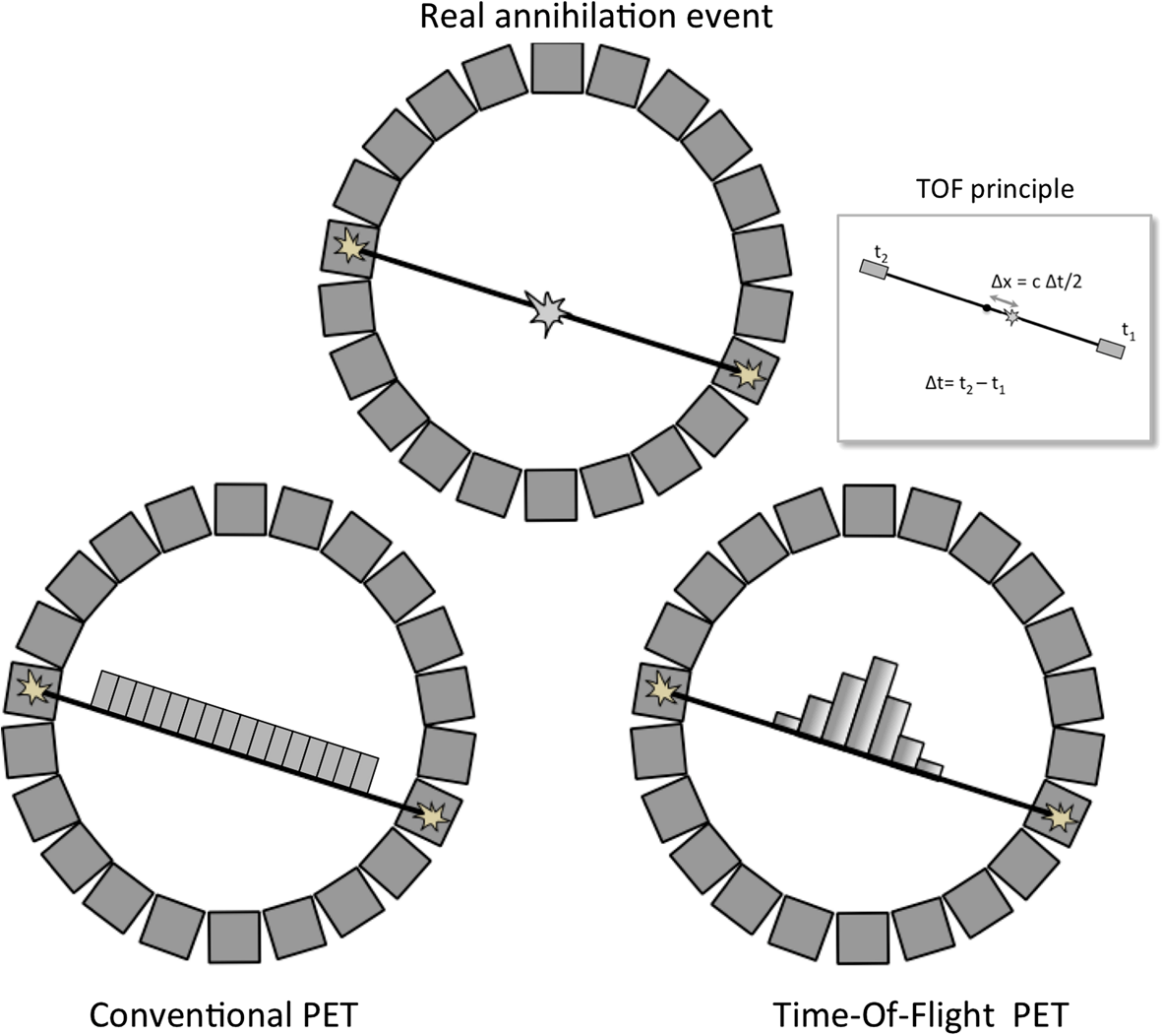
Compared to conventional PET, the estimated time-of-flight difference (*Δ* *t*) between the arrival times of photons on both detectors in TOF-PET allows localization (with a certain probability) of the point of annihilation on the line of response. In TOF-PET, the distance to the origin of scanner (*Δ* *x*) is proportional to the TOF difference via the relation: *Δ* *t*: $\Delta x = \frac {c \Delta t}{2}$, where *c* is the speed of light. *t*_1_ is the arrival time on the first detector, and *t*_2_ is the arrival time on the second detector

Positron emission tomography (PET) is based on the principle of opposed 511-keV photons originating from the annihilation of emitted positron with a nearby electron. In conventional PET coincidence electronics are used to determine along which line of response (LOR) an annihilation has occurred. Time-of-flight (TOF)-PET goes one step further, and we try to determine approximately the position of annihilation along the line of annihilation (Fig. 1) using the measured difference in arrival times.

The principle of TOF has been proposed in the early days of PET technology (Fig. 1). It has resulted in different developments of prototypes during the 1980s [1–7]. These systems used barium fluoride (BaF_2_) or cesium fluoride (CsF) as a scintillator. For complete systems, timing resolutions around 500 ps were obtained. Due to the limited stopping power of the used scintillators, these systems had limited spatial resolution and sensitivity. Therefore, they were not competitive with the high-density scintillator bismuth germanate (BGO)-based systems, which were developed in the same period. The major technology used were block detectors based on pixelated BGO with light sharing to large photomultiplier tubes (PMTs). These had better spatial resolution than the systems based on CsF or BaF_2_, which used one-to-one coupling on PMTs (except the SuperPET systems from TerPogossian in late 1980s based on BaF_2_). During the 1990s, the developments on TOF technology were limited and BGO was the dominant scintillator for PET systems.

An important component in the revival of TOF developments during the last century was the availability of lutetium oxyorthosilicate (LSO). This scintillator has good timing resolution and has also excellent specifications for stopping power and energy resolution. Together with the availability of fast photomultiplier tubes and improved electronics, this has stimulated the development of new TOF-PET systems [8, 9]. The required calibration techniques were further developed, and iterative reconstruction algorithms were modified to incorporate TOF-specific corrections. Sufficient computing power for image reconstruction is now achieved using parallel processing on small computer clusters. Besides LSO (patented by Siemens) also lutetium-yttrium oxyorthosilicate (LYSO) [10] (with similar properties as LSO) was introduced in the early 2000s. The combination of these developments has led to the first commercially available TOF-PET scanner [11, 12] in 2006, and several other commercial systems have recently become available [13, 14].

The main motivation for TOF-PET has always been the potential image quality improvement or reduction in image acquisition time of TOF-PET [9, 15, 16]. This gain is related to the object size, and the largest gain can be expected in heavy patients, which suffer most from poor image quality. The gain has been mentioned and quantified in several studies [12, 17]. Using simulated and measured data, different papers have more recently shown the improved image quality [18–20], especially for heavy patients with lower contrast lesions [21], but also the dependency of the gain on the distribution and the location in the body [22]. TOF-PET can be easily compared with PET data for the same study as the TOF information can be ignored during reconstruction, modeling in the reconstruction (point spread function (PSF) and/or TOF) can be turned off. For reconstruction of the measured data, several options are available ranging from very fast, approximate methods like rebinning [23–25], methods based on a limited number of histoprojections [26], or histoimages and computationally intensive listmode reconstruction methods with minimal approximations [27]. Further progress on the readout and electronics is still being made. Scintillators like lanthanum bromide, LaBr_3_, and improved light detectors (silicon photomultipliers (SiPMs)) have promising characteristics, that can lead to TOF-PET systems with timing resolutions below 400 ps [28–30].

The goal of this review is to describe the recent developments (after 2000) on TOF-PET. The paper reviews the different clinical and prototype systems and the recent developments in image reconstruction and correction techniques. Afterwards, an overview of the papers predicting and evaluating the image quality gain is given. The final part of the review illustrates the potential of TOF-PET for new applications.

## Review

### TOF-PET systems

**Fig. 2:**
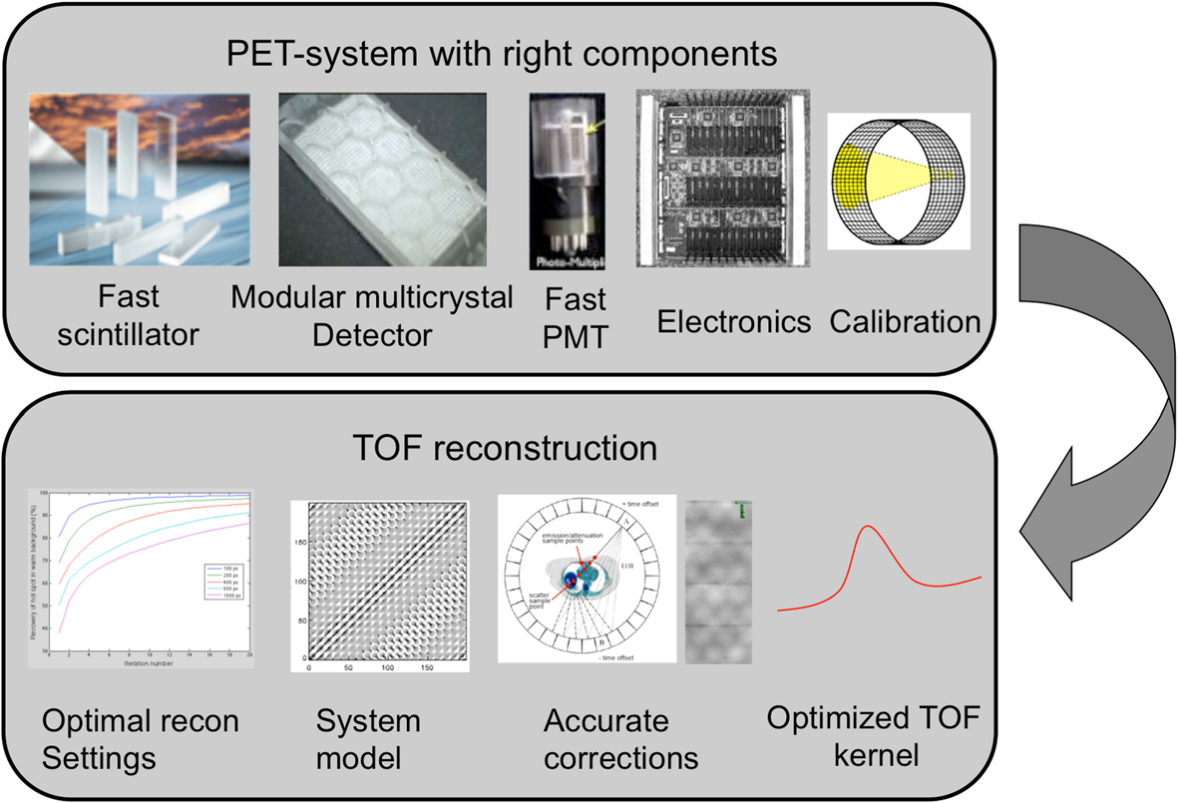
A clinical TOF-PET scanner is a well-balanced combination of fast scintillators, readout hardware, and accurate reconstruction and corrections

The most current clinical TOF-PET-CT systems have a time of flight resolution in the range of 500–600 ps. This timing resolution is determined by the different components involved in the detection process: the scintillator, the photomultiplier tubes, and the processing electronics. This is shown in Fig. 2.

The first necessary component is the use of a sufficiently fast scintillator (and preferably sufficiently high stopping power). The current available scintillators (which are available in large quantities) are LSO, LYSO, and LaBr_3_. LuI_3_ and LuAG (Ce or Pr) are new scintillators, which have very promising characteristics for TOF but are now only available in smaller samples, and it will be very challenging to transition these to large-scale quantities [31–34].

The specifications of these scintillators are given in Table 1; the specifications of LuI_3_ are not given because of the early stage of development of this scintillator.

**Table 1:** Properties of scintillators used in TOF systems

Property	LYSO (10 % Y)	LSO	LaBr_3_
Attenuation coefficient (cm ^−1^)	0.86	0.90	0.47
Decay time (ns)	40	40	27
Light output (photons/MeV)	25–30,000	25–30,000	60,000

The second component is a fast PMT with fast rise time, low transit-time spread (TTS), and high quantum efficiency (QE) at the wavelength of the emitted photoelectrons. Also the size, surface of the scintillator, and the light guide (integrated in the detector module) have an important influence on the timing characteristics. Once a sufficiently fast scintillator is used in combination with a fast PMT, it is necessary to avoid degradation of timing accuracy in the electronics processing. A detailed description of the role and relative importance of each of these components are not within the scope of this review but has been described in several other papers [35–39].

#### Clinical TOF-PET/CT and TOF-PET/MRI systems

The different companies in the domain of medical imaging have now all introduced time-of-flight technology in their whole body PET/CT and some in their PET/magnetic resonance imaging (MRI) systems.

The Philips Gemini TF scanner [11] was the first clinical TOF-PET/CT scanner and has been installed (since 2006) at a large number of hospitals. This scanner operates in 3D mode and has the following reported specifications: 11.5 % energy resolution and a timing resolution of 585 ps. The LYSO crystals have a 4 × 4 × 22 mm^3^ size, and the measured spatial resolution of the system (at 1 cm) is 4.7 mm both transversely and axially. The PET sensitivity at the center is 7 cps/kBq (NEMA 2001). The peak noise equivalent count rate (NECR, a global metric, that accounts for the propagation of noise due to scatter and random correction, related to image signal-to-noise) is about 110 kcps (NEMA 2001). The lower energy threshold of this system is 440 keV, and the width of the coincidence window is 6 ns. Compared to other PET systems, this design is based on a continuous light guide and anger logic detection scheme. The timing resolution (at low count rates) of the first version was 550 ps. From a more recent big bore version of this scanner (oriented towards radiotherapy) with improved electronics, timing resolutions were measured at low rates around 500 ps. The transverse Field-Of-View (FOV) of this scanner is 85 cm instead of the conventional 70 cm. The sensitivity is equal, but due to the reduced shielding, the PET peak NECR is reduced to 94 kcps. These systems acquire and reconstruct data in listmode format. The TOF information in the listmode file is stored with an accuracy of 25 ps (TOF bin size 25 ps). This system is also the basis of the new Philips Ingenuity TF PET-CT, which has a reported TOF resolution of 502 ps [40].

Table 2 gives an overview of the specifications of the different TOF-PET/CT systems published in recent studies. These values are based on specifications provided by the companies and the following publications for the GE system [14], the Siemens [13], and the Philips systems [41, 42]. Table 2 also summarizes features of new TOF-PET/CT scanners. Celesteion^TM^ has been recently introduced in the US market (FDA clearance 2014) by Toshiba as a clinical big bore TOF-PET/CT system. The system is based on a combination of lutetium-based scintillator with PMT-based readout. The reported TOF resolution (improved by using shorter crystals) at low count rates at the module level is ∼410 ps [43]. Another most recent TOF-PET/CT system, Vereos Digital, is developed by Philips. It is based on very new digital SiPM (dSiPM) photodetectors [44] developed and put into practice by Philips Digital Photon Counting in 2009. This is the first system with one-to-one coupling of LYSO crystals with the surface of 4 × 4 mm^2^ to dSiPMs of about the same size. This leads to very high count-rate capability and the Vereos reaches the timing resolution of <316 ps. The differences in sensitivity between the presented systems are determined by the crystal length and the length of the axial FOV. Differences in TOF resolution depend on crystal length, electronic front end, time digitizer, detector architecture (panel or block), crystal assembly, crystal surface and reflector, etc. The performance of all systems are quite close to each other with the Siemens Biograph mCT, excelling in sensitivity (due to its longer axial FOV), and the Philips Vereos in TOF resolution (due to its one-to-one coupling and dSiPMs).

**Table 2:** Specifications of commercially available and recently introduced new clinical TOF-PET/CT systems. Not all specifications are already available

	Company				
	Philips	Siemens	GE	Philips	Toshiba
	System name				
	Ingenuity	Biograph	Discovery	Vereos	Celesteion
	TF [40]	mCT [13]	690 [14]	Digital [41]	[43]
Scintillator	LYSO	LSO	LYSO	LYSO	LYSO
Photo-detector	PMT	PMT	PMT	dSiPM	PMT
Crystal size, mm^3^	4 ×4×22	4 ×4×20	4.2 ×6.3×25	4 ×4×19	4 ×4×12
Total crystals	28,336	32,448	13,824	23,040	30,720
Patient bore, cm	71.7	78	70	70	88
Axial length, cm	18	21.8	15.7	16.4	19.6
Resolution, mm					
Transaxial					
at 1 cm/10 cm	4.8/5.1	4.4/4.95	4.7/5.06	4.1/4.5	5.1/5.1
Axial					
at 1 cm/10 cm	4.73/5.23	4.4/5.9	4.74/5.55	3.96/4.3	5.0/5.4
Energy resolution, %	11.1	11.5	12.4	11.1	NA
Lower *E*_thr_, keV	440	435	425	450	NA
Higher *E*_thr_, keV	665	650	650	NA	NA
Scatter fraction, %	36.7	33.2	37	30	42.7
Sensitivity, cps/kBq	7.3	9.7	7.4	5.7	NA
Coincidence window, ns	4.5	4.1	4.9	4	NA
Peak NEC,					
kcps at kBq/mL	124 at 20.3	180 at 28	139 at 29	171 at 50	153 at NA
TOF bin size, ps	25	312	NA	NA	NA
TOF resolution, ps	502	527.5	544.3	316	∼410

NA (Not Available)

The first simultaneous PET/MR (Siemens mMR) was based on APD readout and therefore not capable of TOF [45]. The two other competitors have introduced TOF in their PET/MR scanners [42, 46]. The Philips Ingenuity TF PET/MRI scanner [42] is a clinical scanner already installed at several hospitals. The TOF-PET and the 3T MR are separated with a rotating bed in-between them, and due to this distance and additional shielding of the PMTs, the PET can still work with PMT-based readout. The PET component [47] of this system is based on the Gemini TF. To limit the influence of MR field, the PET component is placed at a relatively large distance. Additional shielding of the system and individual PMTs make it possible to use the existing readout technology in this system with the magnet on. The scanner electronics is based on the electronics of Philips Gemini TF PET/CT [11], but with necessary changes for the performance in the vicinity of strong magnetic fields (3T). The same as Gemini TF, the Ingenuity TF design is based on a continuous light guide and anger logic detection scheme. TOF measurements are also available on the new GE system, the Signa PET/MRI. The readout of this system is based on analog SiPMs and on a pixelated Lutetium-Based Scintillator (LBS) (Table 3).

**Table 3:** Specifications of commercially available TOF-PET/MR systems

	Company	
	Philips	GE
	Ingenuity TF	Signa
	PET/MR [42]	PET/MR
Scintillator	LYSO	LBS
Photo-detector	PMT	SiPM
Scintillator size (mm^3^)	4 ×4×22	4 ×5.3×25
Total detector elements	28,336	20,160
Bore/detector diameter (cm)	70.7/90.3	60/
Axial length (cm)	18	25
Resolution (in mm)		
Transaxial at 1 cm/10 cm (mm)	4.7/5.15	4.2/5.2
Axial at 1 cm/10 cm (mm)	4.6/5.0	5.8/7.1
Energy resolution (%)	11.6	11
Lower energy threshold (keV)	460	425
Scatter fraction (%)	26	43.6 at peak NECR
Sensitivity (cps/kBq)	7.0	21
Coincidence window (ns)	6	4.57
Peak NEC (kcps at kBq/mL)	88.5 at 13.7	210 at 17.5
TOF bin size (ps)	25	NA
TOF resolution (ps)	525	400

NA (Not Available)

#### Prototype systems

Besides clinical systems based on LSO or LYSO, there are also new TOF-PET prototypes developed by research institutions.

##### PMT-based systems

One of the longest ongoing developments is the LaBr_3_ prototype scanner at the University of Pennsylvania. Based on simulation results described in [18], a scanner was designed. This PMT-based scanner is composed of 24 large modules produced by the scintillator manufacturer Saint Gobain. The crystals in this system are 4×4×30 mm^3^ and the system has an axial FOV of 25 cm (60 rings). Preliminary NEMA NU2-2001 measurements were performed with the axial FOV of 19.35 cm, due to the number of available electronic channels and with five instead of six axial PMT rows [48]. The reported system timing resolutions is 375 ps (with upgraded electronics and improved calibration). The highlight output of this scintillator results in an excellent energy resolution of 7.5 % (the system level). This makes it possible to have a lower energy threshold at 470 keV and to reduce the NEMA-2001 scatter fraction to 25 %. The thicker crystals and longer axial FOV of this scanner compensate for the reduced stopping power of LaBr_3_, and the predicted sensitivity (6 cps/kBq) of this system, once the full system electronics are completed, is quite close to the one of commercial systems.

From the same group, an alternative readout with analog SiPMs has been investigated. Recent measurements are done with the 4×4×30 mm^3^ LaBr_3_ crystals coupled with SiPMs optimized for the near-ultraviolet (NUV) scintillation light emission. Such arrangement showed energy resolution of 6.8 % for 511 keV photons and 245 ps TOF resolution [49], demonstrating the potential of one-to-one coupling with solid-state photodetectors.

Another TOF-PET prototype based on multi-anode high-quantum efficiency (33 % at 420 nm) PMTs (MA-PMTs) is build and evaluated at Seoul National University [50]. This system presents 40 detectors arranged in a single-ring geometry. Each detector is a 15×15 array of 3×3×20 mm^3^ L _0.95_ GSO crystals. The measured transverse and axial spatial resolutions at 1 cm offset from the center of the FOV are 2.15 and 2.41 mm Full-Width at Half-Maximum (FWHM), respectively. This multichannel readout has the potential to provide very good timing, but the technology will very likely not become commercially viable, while SiPMs are already entering the market now in the high-end TOF-PET systems.

Currently, a clinical evaluation of the whole-body PoleStar m660 TOF-PET/CT scanner is carried out [51]. The system is based on 3.63×3.63×20 mm^3^ LYSO scintillators arranged into detector modules (14×14 crystals). The detector modules are then combined into the total of 24 detector buckets (2×4 detector modules) forming a four-ring scanner. The timing resolution of PoleStar is ∼434 ps FWHM. The NEMA NU 2-2007 measurements show 3.76 and 3.64 mm FWHM spatial resolution in transverse and axial directions, respectively, at 1 cm offset from the center FOV, the 10.9-cps/kBq sensitivity at the center of the FOV (425–650 keV energy acceptance window) and the 224.6 kcps at 29.0 kBq/mL peak NECR.

These systems all use similar scintillator shapes (conventional pixelated scintillators), as the current systems and do not aim to estimate depth-of-interaction (DOI) information. Several groups have started working on the combined estimation of DOI and TOF. A new four-layer DOI detector for TOF-PET has been proposed by a group in Japan [52]. They have shown that using the DOI information, and using a timing correction dependent on the depth, can improve time resolution. An improvement in timing resolution (measured in combination with fast BaF_2_) from 730 to 477 ps in FWHM was measured and was combined with four-layer DOI information. The ignorance of DOI leads to a degradation of TOF (different path lengths of light photons) and, therefore the extraction of DOI information can be used to improve TOF resolution. A LaBr_3_ PET detector with good TOF resolution and two-level DOI discrimination was constructed in [53]. The single-ended readout of scintillator stacks with various cerium dopant concentrations (including pure cerium bromide (CeBr_3_)) was investigated and timing resolutions in the range of 150–200 ps were obtained in combination with two-level DOI.

##### SiPM-based systems

More recent developments are oriented towards MR-compatible TOF-PET system. The goal of the EU-FP7 Hyperimage and Sublima project (http://cordis.europa.eu/project/rcn/87568_en.html) is to develop a simultaneous whole-body PET-MR system based on a completely different readout scheme with MR-compatible SiPMs and electronics. This readout system should significantly reduce the pile-up effects of current PET systems and also enable TOF measurements. One of the other challenge in this design is the limited space associated with a simultaneous PET-MR. This is accomplished by designing a very compact detector stack. The first version was based on analog SiPM readout. The second version, called Hyperion-IID [54], is a PET insert, which allows simultaneous operation in a clinical MRI scanner, but is based on digital SiPMs. The system has small pixels (with a pitch of 1 mm) and a bore close to 21 cm in diameter (suitable for imaging up to a rabbit). In the best results (trigger level 1), a timing resolution of 260 ps was obtained and benefits of TOF are demonstrated in a small object of 11 cm in diameter.

Also with SiPMs, efforts have been made to estimate DOI and TOF simultaneously. Most efforts have been made on alternative detection systems, like continuous PET detectors with readout (SiPMs) from one or more sides [55]. This enables DOI measurements with the accuracy around 2–4 mm FWHM. Reading out on both sides improves the accuracy of the DOI and makes it possible to correct for DOI-dependent effects in the timing uncertainty.

##### Other detection systems

Alternative TOF-PET systems can be built using other detection mechanisms [56] than scintillators. The resistive plate chamber (RPC) detector is based on the converter-plate principle. These are low-cost detectors with very good timing characteristics. The limitation of these detectors is their low detection efficiency compared to scintillators. This significant disadvantage can be partially compensated by extending the axial FOV as proposed in [57]. The reported coincidence time resolution of these systems is around 300 ps FWHM. The first imaging results showed that the scanner is capable to achieve spatial resolution of 0.4 mm FWHM [58]. There are however also other disadvantages of this technique (like limited scatter rejection), and scintillator-based systems remain the preferred technology.

Some groups are also investigating to use the prompt Cherenkov light [59–61], produced by the absorption of the annihilation photon in the crystal, to improve the measurements of arrival time difference. It has been demonstrated that with low-cost PbF_2_ crystals [59], as well as with PbWO_4_ (PWO) [60], it is possible to reach coincidence resolving time of <100 ps. Additionally, certain Cherenkov detectors intrinsically reject most of the scattered events. That is because a gamma photon must have energy significantlly higher than the binding energy of an electron to be able to free it and produce Cherenkov light by multiple electron scattering. Normally, energy of annihilation photons (511 keV) is enough to produce electrons to overcome the Cherenkov threshold. In contrast, photons that underwent Compton scattering are not able to do it and thus are not detected. Obviously, the ability to reject scattered events highly depends on such properties of the Cherenkov detector as refractive index and atomic number. One of the major challenges is that quite low amount of photons are generated via the gamma absorption process thus leading to poor energy resolution. Therefore, the Cherenkov technique requires the use of very efficient light detectors with high atomic number, suitable refractive index, and good optical transmission properties for visiblelight.

### Reconstruction and corrections for TOF-PET

Once the measured data is available, reconstruction is used to calculate the image given the available data. In a perfect TOF-PET system (TOF resolution $\rightarrow $0, thus TOF kernel to a delta function), image reconstruction would become not necessary due to the fact that the exact (i.e., as exact as physics laws allow) position of any *e*^+^*e*^−^ annihilation point can be calculated from its LOR coordinates and time difference information using the equation shown in Fig. 1. However, in current systems, image reconstruction is still a necessary step to obtain the final image. The additional information provided by the TOF measurement adds extra complexity to the process of image reconstruction. While the first developments in the 1980s were making use of analytical reconstruction techniques, nearly all systems are now using exclusively iterative reconstruction methods. Analytical methods however still have benefits in terms of speed and the potential for more consistent (not dependent on iteration number or distribution) quantification. Iterative methods also suffer from low bias in low-count situations due to the non-negativity constraint which is typical for Maximum Likelihood Expectation Maximization (MLEM) (i.e, image voxels never have negative values due to the multiplicative nature of MLEM and the fact that the initial image is not negative). The main reason to use iterative methods is their better noise behavior when compared to FBP. Current computing power enables the use of the computationally demanding listmode reconstruction methods, but it remains a challenge to keep track with the faster acquisition times (down to 1 min per bed position). Rebinning techniques are therefore still interesting to limit the data size and to reduce the reconstruction time.

#### Data formats

The most natural format for TOF-PET is to store the data as a list of events, with the necessary details of each event (called listmode data). This allows to use 3D listmode TOF MLEM. Normalization, dead time, and attenuation correction [62] can be done using multiplicative correction factors. Additive correction for scattered and random events is more complex compared to sinogram-based reconstruction. First, a coarse sinogram is created containing a random or scatter distribution. Based on this distribution, an additive factor (representing the random or scatter fraction per listmode event) is calculated and then added to the forward projection of the current estimate. Listmode reconstruction algorithm is quite slow, as it processes event per event, and it results in a reconstruction time dependent on the number of events recorded. Because the listmode events are not geometrically sorted, it is almost necessary to use on-the-fly calculation for forward and back projection, which is significantly slower than pre-calculated projection and back projection. To make the slow reconstruction acceptable in clinical routine, commercial systems use a parallel version implemented on a small cluster with typically 10–20 nodes [62]. Furthermore, speedup factors are obtained by integrating only along a short segment of the LOR around the most likely point. Kernel truncation can increase the speed of reconstruction up to a factor two but should be done carefully as contrast loss can be introduced by too much truncation [63].

During a typical 3D multi-bed whole-body PET study, with the most recent systems, 100–1000 million coincidences, are acquired. If each event needs to be stored in the listmode data set, one has to try to store each event as compact as possible. Even a compact representation (e.g., 4 bytes per event) will easily lead to files of several gigabytes per patient study. To store this information, only a limited number of bits is available. TOF difference is therefore typically stored as multiples of a certain minimal unit, e.g., in the Gemini TF, 25 ps units are used, which is surely fine enough considering that the TOF resolution is 500–550 ps. Eight bits leads therefore to a range of 6.4 ns, adequate to cover the entire FOV. These data are preserved and are reconstructed using a fully 3D relaxed listmode ordered subset expectation maximization (OSEM) reconstruction algorithm [64].

Another approach is to create TOF sinograms, which are sinograms with an additional dimension for the TOF information. The Siemens scanner uses 13 different TOF sinograms (312 ps per sinogram). Each projection element in a conventional 3D PET scanner corresponds to a radial distance, a transverse angle, and an axial detector pair (or axial position + angle). Compared to the PET format, the TOF bin adds another dimension to this data set. These relatively large bins cause some accuracy loss, but the use of 3D TOF sinograms has also advantages: it results in a fixed storage size per study and is the basis format for advanced rebinning techniques (like Fourier rebinning (FORE)). For gated and dynamic studies, the sinogram format is however a significant drawback as the sinogram size multiplies with the number of phases or time frames. In listmode data, this requires just an additional field in the listmode format.

To further reduce the size of the data sets, several papers have also described hybrid reconstruction methods. These methods reduce the data to 2D data, which are then reconstructed by fast 2D reconstruction algorithms. TOF-PET data contain redundant information, and several methods can be used to reduce the dimension of the datasets. One can re-bin the data into a lower dimensional format: 2D TOF, 3D non-TOF, or 2D non-TOF [65]. Even rebinning to non-TOF sinograms, and using the conventional PET reconstruction, can be done with a significant preservation of the TOF gain in signal-to-noise ratio (SNR) [65]. The SSRB-TOF [23] is a very simple method, which uses the TOF information to determine the most likely slice and reduces the number of axial angles due to localized nature of TOF. The TOF information can also be used to reduce the number of transverse angles and mashes the 2D data with a limited loss in information [25]. This rebinning can be done on-the-fly (without the intermediate step of 3D sinograms) and is therefore also interesting to implement in hardware [66]. These methods can make use of Fourier-based projection techniques [67], which further reduce the reconstruction time and open up the possibility of hardware-based image reconstruction. Other approaches like exact or approximate FORE-TOF [24, 68, 69] require sorting of listmode events into sinograms before rebinning.

#### TOF-specific corrections

To transfer the additional information measured by TOF-PET into effective image quality gains, it is necessary to have an accurate modeling of the system and to implement different corrections specific for time-of-flight. These are also needed to obtain quantitative images. The corrections are still being optimized as more experience is obtained with these scanners in clinical routine.

There are two different effects. The first effect that needs to be corrected for is timing offset. Assume we have a point source in the center of the scanner and we consider coincident crystal pairs. As this point source is at equal distance to the two crystals (involved in a coincidence detection), the average TOF difference between two coincident pairs should therefore be equal to zero. However, there are differences in time delays due to the PMTs coming from delays and variations in PMT gain and electronic triggering. Additionally, the relative position of each crystal from the center of a PMT will introduce a certain delay. Similar effects take place in other photodetectors as well. For instance, there is always some uncertainty in timing with scintillators coupled with SiPMs. The nature of these uncertainties is beyond the scope of this paper, but normally they depend on such factors as applied bias voltage, temperature, and triggering threshold of SiPMs. The goal of TOF-offset correction is to minimize these effects. These TOF offset corrections are typically implemented as crystal-based corrections.

**Fig. 3:**
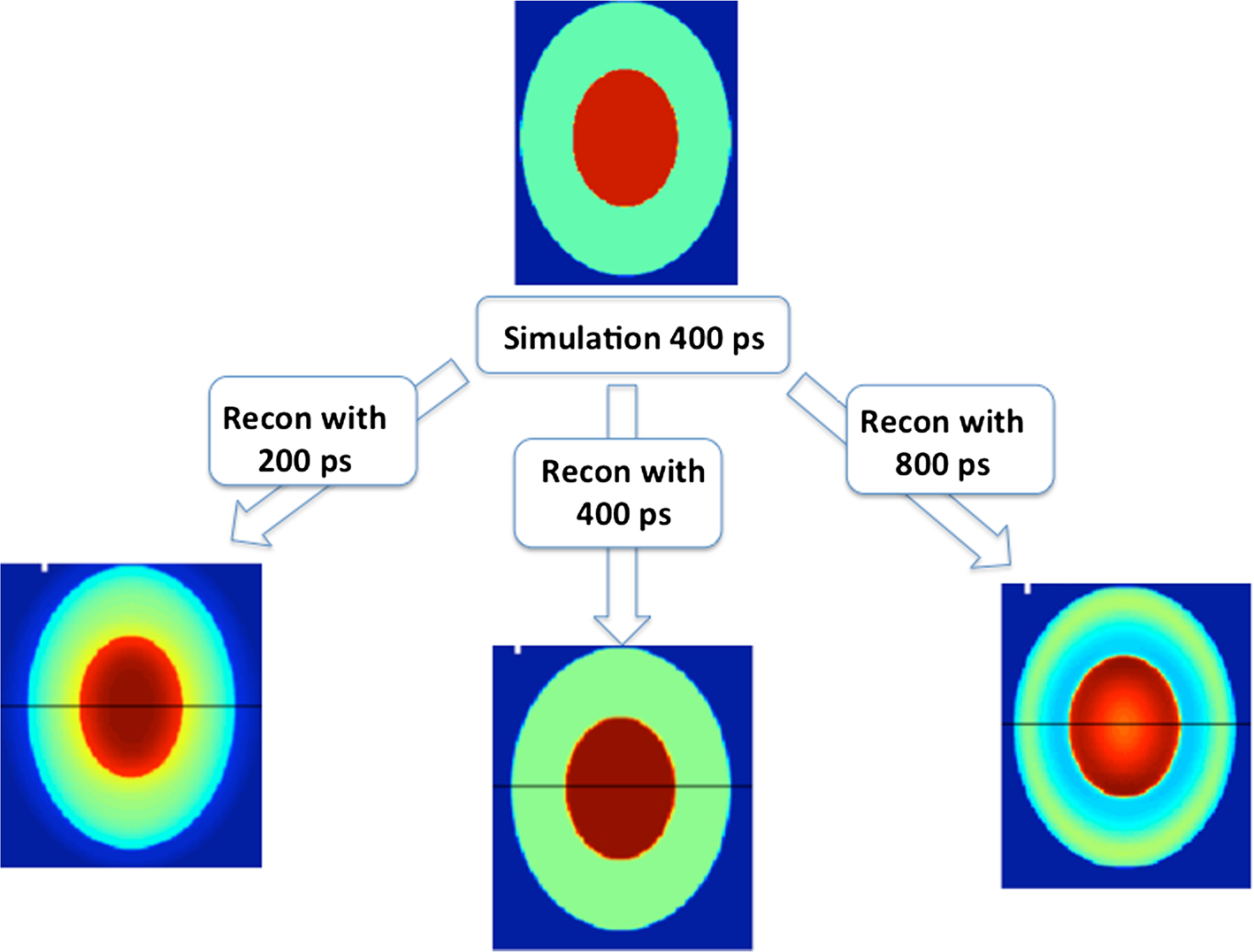
Using a wrong TOF kernel in reconstruction can lead to undershoot (too small TOF kernel) or overshoot (too wide TOF kernel) in the reconstructed image

The second difficulty is the TOF kernel used in image reconstruction. This kernel should represent the probability distribution of the annihilation point along the TOF direction for a zero time difference. Typically, it is assumed to be a Gaussian distribution. When reconstructing images from data, one should always set the kernel width equal to the TOF resolution of the PET scanner with which this data is acquired. An illustration of what happens when a wrong kernel is used in reconstruction is given in Fig. 3. Here, the data is simulated with a PET scanner which has 400 ps TOF resolution. The top image is the true image of the scanned object. Figure 3 illustrates that if the chosen kernel width is smaller (200 ps) than the TOF resolution of the system, the reconstructed activity tends to concentrate at the center of the imaged object (left image). Alternatively, if the chosen kernel width is bigger (800 ps) than the TOF resolution, the reconstructed activity concentrates towards the edge of the object (right image). Correctly chosen kernel width (400 ps in this case) results in reconstructed activity distribution that is as close as possible to the real one (center image at the bottom).

##### TOF offset correction

The timing offset between coincidence pairs must be known accurately to be able to reconstruct correctly. Coincidence time alignment is also performed in conventional PET scanners to correct for variations in propagation time. The accuracy needed for TOF-PET is of course higher, as this information is used in reconstruction to center the Gaussian profile in forward and back projection.

**Fig. 4:**
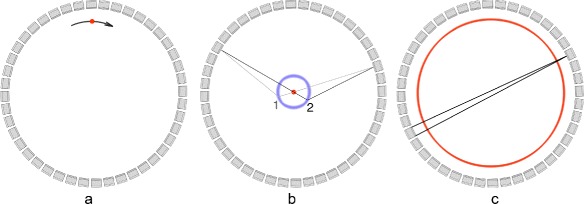
The acquisition methods for obtaining TOF offset calibration data. **a** Using a rotating line source. An emission from the line source (*red*) is detected by crystal pairs connecting via straight line through the point. **b** Using a scattering phantom (indicated in *blue*) and a point source. An emission from point source (*red*) is detected after scattering at *1* or *2*. **c** Using an annulus shape source. The set of close LORs (in *black*) is averaged and used to calculate the average offset per detector pixel

A time alignment probe (a radioactive source embedded in a plastic scintillator coupled with a PMT) has been proposed for this purpose [70], but this requires a modification of the existing systems and has therefore not been implemented on commercial systems. Other methods make use of positron sources that are introduced in the FOV. To be able to calculate the offset, the exact location of the source needs to be known. Therefore, sources like point, line, plane, or uniform cylindrical sources are used. Different methods have been proposed to measure these offsets. A radioactive line source rotating close to the crystals irradiates all crystal pairs after one rotation (Fig. 4a) [71]. In conventional PET scanners, this was easy to implement as rotating line sources were used for transmission scanning, but it would require modifications to the PET-CT scanner. An alternative approach is to use a point source in a scattering object (Fig. 4b) [71]. This object generates coincidence events between pixel pairs, which are not on the same line as the point. This technique is used to determine timing offsets in the Philips Gemini TF.

The authors of [72] assessed the influence of local TOF kernel miscalibrations on the contrast to noise ratio and proposed a third method for TOF offset calibration. They investigated the TOF offset calibration on a clinical system using a stationary solid source, with an annulus shape and a diameter only a few centimeters smaller than the diameter of the FOV (Fig. 4c). After averaging close LORs, the offset per detector pixel was calculated. They found that performance of the clinical system would benefit from a regular calibration for TOF offset. In their study, they also assessed that an underestimation of the TOF kernel of the system has a higher probability for deteriorating image quality than an overestimation.

Due to the high number of crystal pairs (several millions) in a PET scanner, timing offsets are determined per crystal. A timing offset for each detector crystal pixel is obtained by calculating the average offset values of each crystal pixel with the opposing pixels. This corrects for trigger variations, crystal differences, and for photomultiplier (PMT) timing differences. The final result is a look-up table with offsets per detector crystal. This correction is then introduced in the listmode data or offline in image reconstruction by adding the offsets of each of the crystals in the pair.

In a paper by the University of Pennsylvania [73], a timing calibration technique is presented that eliminates the need for a specialized data acquisition and can even enable retroactive calibration of datasets where the calibration is missing. This method works on the patient data directly. It can in principle be updated daily and can therefore be used to compensate for shifts in the calibration, to minimize the possibilities of image quality degradation.

One of the questions remaining is, how often this calibration has to be repeated and how a quality control method for this effect has to be established and integrated in conventional NEMA routines. Recent measurements by our group have shown significant drifts on one module (200–300 ps offsets) after 1 year of using the Philips Gemini TF. These were easy to correct but not detected by the company software. The reason are slow drifts in PMT gains that also affect timing and therefore some systems tweak the calibration on a daily basis (only gains on PMTs) and then measure and report the energy and timing resolution of a point source. The effect of drifting offsets on contrast recovery and image quality has been studied by simulations in [74]. They introduced random offsets in 200 ps TOF datasets and showed the reduction in contrast recovery due to these offsets.

Concerning SiPMs, similar to PMTs, they require a periodic TOF offset calibration. However, behavior of the gain drifts for SiPM photodetectors at the system level is not yet published.

##### TOF kernel modeling

One of the other difficulties in reconstruction is the modeling of the right kernel in the forward and back projection. It has been reported [11] that the kernel width depends on count rate, and different papers have shown that wrong kernels lead to incorrect reconstructions [22, 75, 76]. Too small kernels lead to reconstructions with reduced contrast and unrestored edges. Increased contrast can be obtained with larger TOF kernels but at the expense of background uniformity. Reconstructions of simulated data clearly indicate, that only the correct kernel should be used to reconstruct the data. Therefore, being able to estimate the TOF kernel from the data itself [75] or from additional measurements can be useful.

The effect seems however less pronounced in measured data, where a relatively wide range of kernels result in similar contrast recovery [63]. The current approach to control this effect in the Philips Gemini TF is to use a kernel, which depends on the measured singles count rate. Recent data from the new Siemens system [13] with advanced Pico electronics show a very minor effect of the count rate on timing resolution (less than 50 ps over the full relevant range), these are however measured using a line source, which has a much higher coincidence to singles fraction than the point source with two extra cylinders.

##### Scatter and random correction

Nowadays, scatter correction is calculated on most systems using fast Monte Carlo techniques based on the emission distribution and a density map obtained from the CT data. The standard single scatter simulation (SSS) algorithm [77] simulates the scatter distribution based on a first reconstruction of the emission distribution and the density map. This scatter estimate is then used to correct the next iteration for scatter (using an additive factor in iterative reconstruction). In contrast to the random distribution, the scatter distribution does depend on the TOF difference as scattered events are originating from the same decay (or annihilation). A dedicated TOF-SSS algorithm [78, 79] uses the same scatter distribution as calculated by SSS and does not sum this distribution into projection data but blurs it by the expected TOF kernel. This scatter distribution is then added to the forward projection in iterative reconstruction (given the TOF information of the event).

The randoms can be estimated using the conventional delayed window method. To determine a random fraction per measured event, randoms (measured by the delayed coincidence window method) are first stored on a coarse grid. The correction is then performed in the same way as the scatter. Random events originate from different independent decays. Therefore, measured TOF difference of such events does not give any information about the source position. For this reason, there is not a special random correction method for TOF data.

##### Attenuation correction

Just as the correction for random events, the implementation of attenuation correction is not TOF dependent. When an attenuation map for attenuation correction in PET image reconstruction is absent, it is possible to simultaneously reconstruct emission and transmission images from PET emission data only. The maximum likelihood reconstruction of activity and attenuation (MLAA) is an iterative reconstruction algorithm that uses interleaved updating of emission and attenuation maps [80]. The method, as originally proposed, did not use TOF information and, therefore, the algorithm had a non-unique solution. Consequently, the MLAA reconstruction algorithm suffers from cross-talk between attenuation and emission images. The MLAA method (with or without TOF) is limited to tracers, which have uptake in the full object, because attenuation values can only be recovered in regions with sufficient emission.

However, the use of TOF information can reduce the cross-talk as it is shown theoretically by [81] and experimentally by [82]. The authors of [81] proved that the TOF information stabilizes the joint estimation problem of MLAA, and that the attenuation sinogram in TOF-PET can be determined up to a constant scaling factor. In image space, this means that emission maps show a global scaling factor, whereas attenuation maps have a position-dependent scaling. It was suggested to solve the scaling problem in TOF MLAA by incorporating prior knowledge of the attenuation values by using for instance an external transmission source. Additionally, using a transmission source solves the restriction of MLAA to regions of sufficient tracer uptake. The MLAA method in TOF-PET, with additional information about the attenuation image from an external positron source, was investigated by [83, 84] and (Mollet P, Vandenberghe S: Comparison of transmission- and emission-based attenuation correction for 1091 TOF-PET/MRI, unpublished). The authors of [83] use a rotating rod source on a TOF-PET/CT scanner to refine the CT-based attenuation map. The papers (Mollet P, Vandenberghe S: Comparison of transmission- and emission-based attenuation correction for 1091 TOF-PET/MRI, unpublished) and [84] show a simulation study and patient study, respectively, where the authors use an annulus-shaped source on a TOF-PET/MR system. The simulation study (Mollet P, Vandenberghe S: Comparison of transmission- and emission-based attenuation correction for 1091 TOF-PET/MRI, unpublished) showed that the TOF information could also be used to discern events originating from the patient and events originating from the transmission source in a simultaneous acquisition. They showed that the extraction of transmission events based on TOF information allows accounting for the scaling problem of MLAA even better, by reconstructing an attenuation map prior to MLAA reconstruction.

### Image quality gain with TOF-PET

#### Factors responsible for the TOF gain

**Fig. 5:**
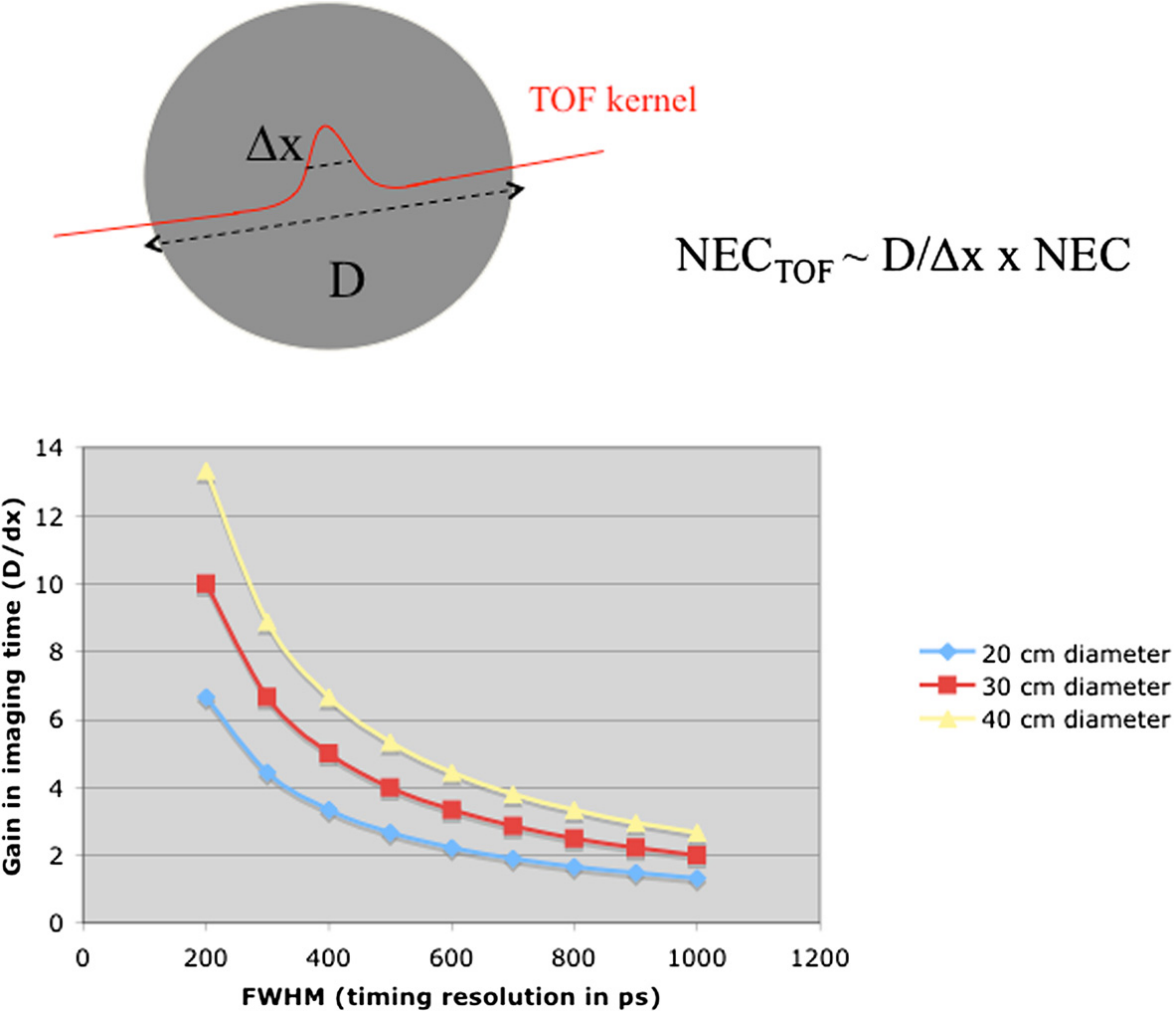
TOF gain is proportional to the ratio of object size to the spatial TOF kernel width. This calculation [4] assumes analytical reconstruction and a simple uniform cylinder of activity

The use of the TOF difference in reconstruction reduces the noise propagation along the LOR during forward and back projection of the data. The reduced noise propagation is related to the physical extent of the TOF kernel and the object size as shown in Fig. 5. A recent paper [54] shows gains due to the TOF in even 11-cm-big objects.

Since the early papers describing the potential gain of TOF-PET, several studies were performed to validate this gain. The effective sensitivity gain was described in [4] as the ratio between the object size *D* and the spatial FWHM of the TOF kernel △*x*. Another paper (taking into account image reconstruction) by Tomitani predicts a smaller gain of ${D}\over {1.6 \triangle x}$. A recent lesion detectability study [20] using simulated data found the gain in non-pre whitening SNR (NPW-SNR) to correlate well with the gain predicted by Tomitani.

As the SNR is proportional to the square root of the number of detected counts, there will be an improvement in SNR equal to the square root of the gain factor. Both relationships describe the gain of noise reduction by TOF on true coincidences only. Both scatter and randoms [85] have also the same factor, due to the reduced noise propagation caused by the localized nature of the TOF kernel. The effect of TOF is however more complex. First of all, the good timing resolution reduces significantly the negative effect of randoms on the NEC performance.

The better the TOF resolution, the better one can discriminate randoms from true events. That is because random events with a time difference often result in a position outside the imaged object. Therefore, these events do not contribute to image noise in the object because they are not being forward and back projected over this object. A TOF-PET scanner has an effective coincidence window width, which depends on the object size. For scattered events, this is only a minor effect as the TOF difference of most scattered events (which originate from the same positron decay) will place the event back into the object.

Currently, systems are often compared using the global NEC metric, which takes the positive effect of the trues and the propagation of noise from removed random and scatter event into account. The additional TOF information is not taken into account into this metric. A modified version taking TOF into account has been proposed [85]. Before it can be included into NEMA guidelines, more validation and a standardized measurement technique are necessary. A more extensive description of introduction of TOF gain into NEC can be found in [86].

#### How to evaluate TOF gain?

The combination of the different effects described above makes it difficult to determine one single TOF gain for a specific scanner. The gain will depend on the number of randoms and the count rate. When using iterative reconstruction methods, a straightforward evaluation becomes even more complex due to the non-linearity of iterative methods and the different convergence of TOF and non-TOF reconstruction. In most objects, TOF reconstruction converges much faster than PET reconstruction. Therefore, less iterations are needed to obtain the same contrast. This effect seems more pronounced for smaller lesions. Object-dependent convergence can make it even impossible to determine matching iterations between TOF and non-TOF reconstruction for the whole image.

The gain can be quantified using a variety of methods. First of all, one can choose between simulated or measured data, where simulated data and measured phantom studies have the advantage of knowing the true distribution. Another advantage of simulations is the controlled environment: one can select either the true, scattered, and random coincidences from the dataset, which allows separation of the different effects. The exact TOF kernel is also known and effects of count rate can be avoided. Measurements on the other hand are more realistic. A quantitative comparison of measured data reconstructed with and without TOF also requires optimal corrections for both reconstructions. Secondary effects like kernel widening (due to increasing count rate) can make it difficult to evaluate pure TOF effects. Mixtures between simulated data and measured data [21, 87, 88] can also serve as interesting evaluation data sets. The general truth (the activity and position of the lesion) is known, and the background is more realistic with regards to structure and attenuation.

The evaluated figure of merit also varies between different studies. Relatively, simple evaluation on single phantoms can be done using contrast-noise curves, more complex evaluation like NPW-SNR, channelized hotelling SNR (CHO-SNR), and observer studies require multiple datasets. The evaluation on patient data is more complex as the true distribution is not known, background is not uniform, and noise is difficult to evaluate. Therefore, relative measures as the change in contrast are used for patient studies. Observer studies require a large number of datasets and extensive reviewing time by nuclear medicine physicians and/or radiologists.

#### Evaluation on simulated or measured phantoms

Most recent papers focused on the gain obtained with iterative reconstruction methods. The first studies were based on simulated data (modeling a LaBr_3_ system) as no clinical scanner was available. In [89], data were simulated with different timing resolutions (from 300 ps to non-TOF-PET). The detectability (based on NPW-SNR) was also improving with better TOF. These results were obtained using hot spots of different sizes in a uniform cylinder of 27 cm in diameter. It was shown that contrast recovery improves with better timing resolution.

This simulation work was extended towards different phantoms (20, 27, and 35 cm diameter). The contrast recovery coefficient (CRC) versus noise results indicate a gain, equal to the ratio between the object size *D*, and the spatial FWHM of the TOF kernel △*x*. The NPW-SNR results correlate better with the smaller gain predicted by Tomitani.

In [90], measurements with a 20- and 35-cm-diameter phantom were performed on the first Gemini TF. It was shown that TOF and PET reconstructions of the small phantom at equal NEC are very similar, while they were quite different for the larger phantom. Small 1-cm spheres were difficult to detect on the PET images, while clearly visible on TOF-PET reconstructions. The CHO-SNR was also clearly higher for TOF at the same NEC rates.

These studies all used uniform phantoms to evaluate the gain. A more recent study [91] used a more realistic anthropomorphic phantom. This was scanned multiple times on a Siemens Biograph system, and different spherical lesions were inserted. The goal of the study was to evaluate the relative gain of accurate point spread function modeling (called PSF) and TOF reconstruction and a combination of both. Because of different convergence properties, the optimal settings for the four different algorithms (OSEM, OSEM + PSF, OSEM + TOF, and OSEM + PSF + TOF) needs to be determined first. The optimal combination of iteration number and post-filter was determined using LROC analysis. The lowest detection probability was obtained without modeling, the inclusion of TOF resulted in a somewhat larger improvement compared to system modeling. The combination of both resulted in the highest improvement.

#### Evaluation on patient studies

In [12], the change in contrast was validated using phantom studies and patient data. It was shown that there was a significant TOF gain. However, this gain is difficult to quantify into one single factor, because TOF improves imaging performance of a PET scanner in several ways comparing to non-TOF-PET:

- Increases effective sensitivity;
- Increases rate of reconstruction algorithm convergence;
- Makes convergence more uniform;
- Improves contrast recovery at matched noise; and
- Benefits even greater for larger patients.

The evaluation on patient data was performed by matching PET and TOF-PET reconstructions, using iterations with equal noise levels in the liver. The gain in contrast was higher for heavier patients. The average gains increased from 15 (light-weight patients) to 40 % (140-kg patients). Results [21] used a mixture of normal patients and simulated hot spots to enable efficient observer studies. These hot spots were inserted at different locations in the body (liver, lung). The data were processed using mathematical observers. TOF delivered an increase in CHO-SNR of 7.7 % in the liver and and a higher gain of 14.3 % in the lungs. The gain was also higher for patients with BMI larger than 30. The same data were also presented [87] to experienced human observers, and similar findings were reported. This work was extended in a more recent study [88]. In this study, the accuracy (bias) and precision of embedded lesions in the liver and lung of six patients was evaluated for TOF and non-TOF PET. In the lung, an increase of the average uptake with 50 % and in the liver with 20 % was obtained by using TOF. There was also a reduction in variability.

In the paper [91] mentioned before, two patient scans were also processed. It was shown that improved SNR could be obtained at lower noise levels. Visual improvements for lesions in the liver were also noticeable. Evidence of improved SNR is presented in the following research [12, 17].

The improvement in clinical reality is now mostly used to reduce the imaging time. Other options are the reduction of injected dose. Both will result in a lower cost per study. The final goal of improvement of image quality in oncology is to improve the detection of tumors. For follow-up studies, it is important to improve the quantification accuracy. To better evaluate the effective gain, multicenter studies should be performed. These can be performed using TOF-PET data reconstructed with and without TOF information. The same should be done for cardiac and brain studies, as the gain will be different for these areas.

#### Use of the gain in clinical scans

One can benefit in different ways from the time-of-flight information in a daily clinical environment. By keeping the acquisition time constant, time-of-flight information leads to increased contrast recovery due to faster convergence. This is especially noticed for the smallest lesions. One can also use the information to reduce the acquisition time resulting in a higher patient throughput per day. Finally, it is also possible to limit the injected dose. This will result in a reduced cost per scan (especially important for sites not owning a cyclotron) and less radiation dose for the patient. This becomes an important concern as the relative dose of PET in PET-CT has been increased by the introduction of low-dose CT scans.

To obtain more consistent image quality over the patient population, a patient-dependent protocol acquisition is recommendable. This should be dependent on the weight, BMI, or torso size of the patient and can include both injected dose and acquisition time. Although TOF is showing a larger gain in heavier patients, one should take into account that the effect of attenuation remains a major factor in the image quality loss in interior regions. A recent protocol has suggested a range of acquisition times from 1 min per bed position (for light patients) up to 3 min for heavier patients [92]. A typical protocol will be based on the body mass index: 90 s for a BMI below 30, 2 min for a BMI in the range of 30–35, and 3 min for patients with a BMI larger than 35.

TOF-PET has further evolved during the last years towards better TOF resolution (300–400 ps). The goal is to do this with limited loss in sensitivity and spatial resolution. The progress will be gradual as all the different components need to improve further to obtain better TOF resolution. In clinical reality, this should result in better detection and improved quantitative accuracy of small lesions. Cold defects in cardiac imaging may also benefit from the improvement with TOF, but we can not directly infer the degree of improvement from studies of hot lesions.The expected improvements by TOF will lead to a better accuracy of quantification, and the improved detectability of smaller hot spots. Therefore, it should have a positive effect on patient management. A good example is the follow-up of the effect of radio/chemotherapy using quantitative PET data, which will become more accurate and may therefore be used more frequently.

#### Other advantages of TOF

**Fig. 6:**
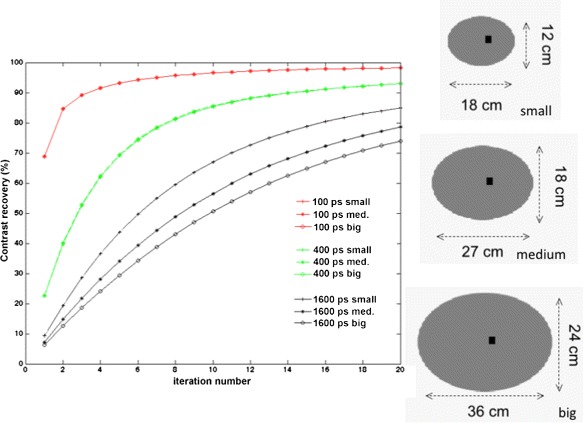
The use of better TOF information leads to reduced dependency of convergence on the object size

Besides the conventional image quality gain, different researchers have also reported on other benefits due to the local nature of TOF. In general, the use of TOF reduces the effect of object size (and surrounding activity) on the convergence. This is shown in Fig. 6.

There is also a more uniform convergence (less dependent on surrounding activity) due to TOF. This improved convergence by iterative reconstruction is especially strong in cold regions like the lung, which was shown using simulated data [93] and measured data [94]. This can be explained by the limited effect of surrounding activity due to the short range kernel. Higher gain in image quality was also predicted in the cold regions by a detailed study [22]. These results also help to explain the results reported in [88] in which the uptake of embedded lesions in the lung is lower than those embedded in the liver. It also illustrates the challenge of characterizing the benefit of TOF on real data with simple metrics as those proposed in [4, 85].

In general, TOF also helps to improve consistency of reconstruction and makes it less prone to inconsistencies between emission data and corrections [95]. This applies to inconsistent normalization, absence of scatter correction, and mismatched attenuation correction (in PET-CT often present in the case of respiratory motion). One can also use this to iteratively solve for efficiency factors from an arbitrary distribution [96]. This property also has advantages in PET/MR, where attenuation correction is only approximately known. Table 4 summarizes the impact of TOF imaging on the different levels.

**Table 4:** Impact of TOF imaging on the different levels

PET performance	Image reconstruction	Image quality	Clinical performance
Reduced effect	Reduced impact of	Reduced image	Reduced acquisition
of randoms	small errors in	noise	time or dose
	data correction ^b^		
Higher NEC	Better algorithm	Higher SNR ^a^	Gain in heavy
	convergence		patients
	Better convergence	Better small lesions	Improved lesion
	uniformity	quantitative accuracy	detectability
		Better overall image	More accurate
		quality ^a^	quantification

^a^Especially for heavy patients

^b^Inconsistent normalization, absence of scatter correction, and mismatched attenuation correction (e.g., due to motion)

### New applications with TOF-PET

Most publications reported the optimization or gain of TOF for lesion studies with FDG on the existing clinical PET systems. Due to its local properties and higher effective sensitivity, there are also new applications that can benefit from TOF-PET.

#### Local tomography

The potential of local tomography for TOF-PET was illustrated in [97]. They investigated the reconstruction of ROIs (from 1 pixel to 144 mm) using truncated projections. Truncated 2D TOF-PET (700 ps) could be used to reconstruct ROIs of single pixels, which was not possible with 2D PET data. Visually, the images obtained with TOF-PET are much closer to the correct distribution. This property can be interesting if there is only a limited area of interest (e.g., cardiac imaging) and fast reconstruction is necessary. Other applications mentioned in the above reference are local motion compensation.

#### Separation of events based on their origin

**Fig. 7:**
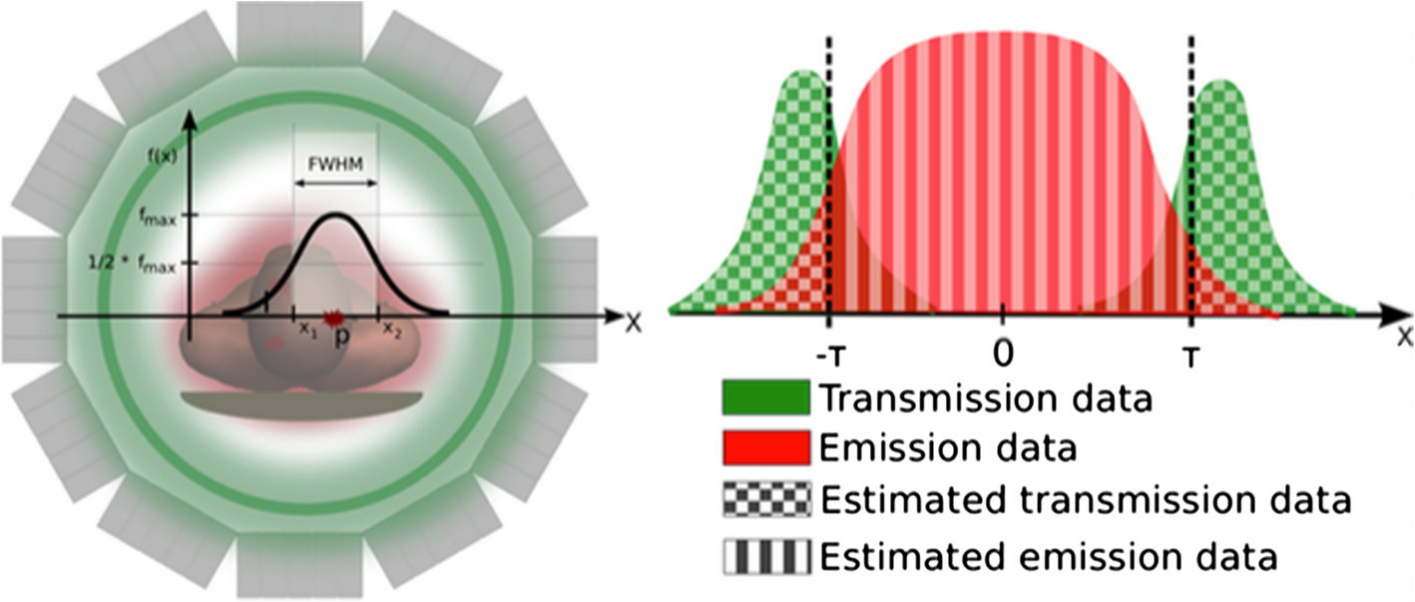
TOF-based extraction of transmission data on a particular LOR for a threshold radius *τ*

The localized nature of TOF-PET also allows to separate events based on their origin in image space. One particular interesting application of this is to distinguish between events, coming from the emission object and from a transmission source [35] (Fig. 7). Once these are separated in image space by 1–2 TOF-FWHM, they should be almost perfectly identifiable. Simultaneous transmission and emission is not useful for current PET-CT scanners, as the transmission data are not measured with 511 keV but are obtained from a scaling of the CT image. For dedicated brain scanners and future PET-MR however, this property may be interesting. Other applications of this property are the separation of two different emission objects well separated in image space: examples of this are the scanning of multiple animals on human systems. Once these are separated by 1 to 2 TOF-FWHM, the listmode dataset can be split into different sets for each animal, and they can all be separately reconstructed (using the same attenuation map!).

#### MR artifact reduction by TOF

Recent simulation [98] and clinical [99] studies have also illustrated that attenuation artifacts due to metal and respiration are less visible in TOF reconstructions. In PET scanners, a normalization scan is used to minimize the effect (on the final reconstructed image) of the different performance of crystals. It was noticed that there was less influence in image space due to errors in these normalization maps when TOF image reconstruction is used [95].

This has now a particular advantage for TOF-PET-MR. Since the advent of PET/MR, great efforts have been made to optimize MR-based attenuation correction. The MR-based attenuation correction methods all suffer from MR-related difficulties, from which some can be reduced when using TOF information. A recent study on the impact of TOF on quantification errors in PET/MR is presented in [100]. The study shows that especially in the lung and bone, TOF PET reduces the influence of MR segmentation errors compared to non-TOF PET. In the same paper, the influence of TOF on metal-susceptibility artifacts is discussed. Their findings are in agreement with [98], where the authors proved with a simulation study that TOF information reduces the influence of metal artifacts in PET images. Furthermore, [100] assessed the influence of TOF on respiratory phase mismatch in PET/MR and for this MR artifact, too, a reduction of bias is observed when using TOF. The authors of [100] foresee that MR-based attenuation correction will improve as TOF resolution increases in the future.

#### MR-less attenuation correction in PET/MR with TOF

**Fig. 8:**
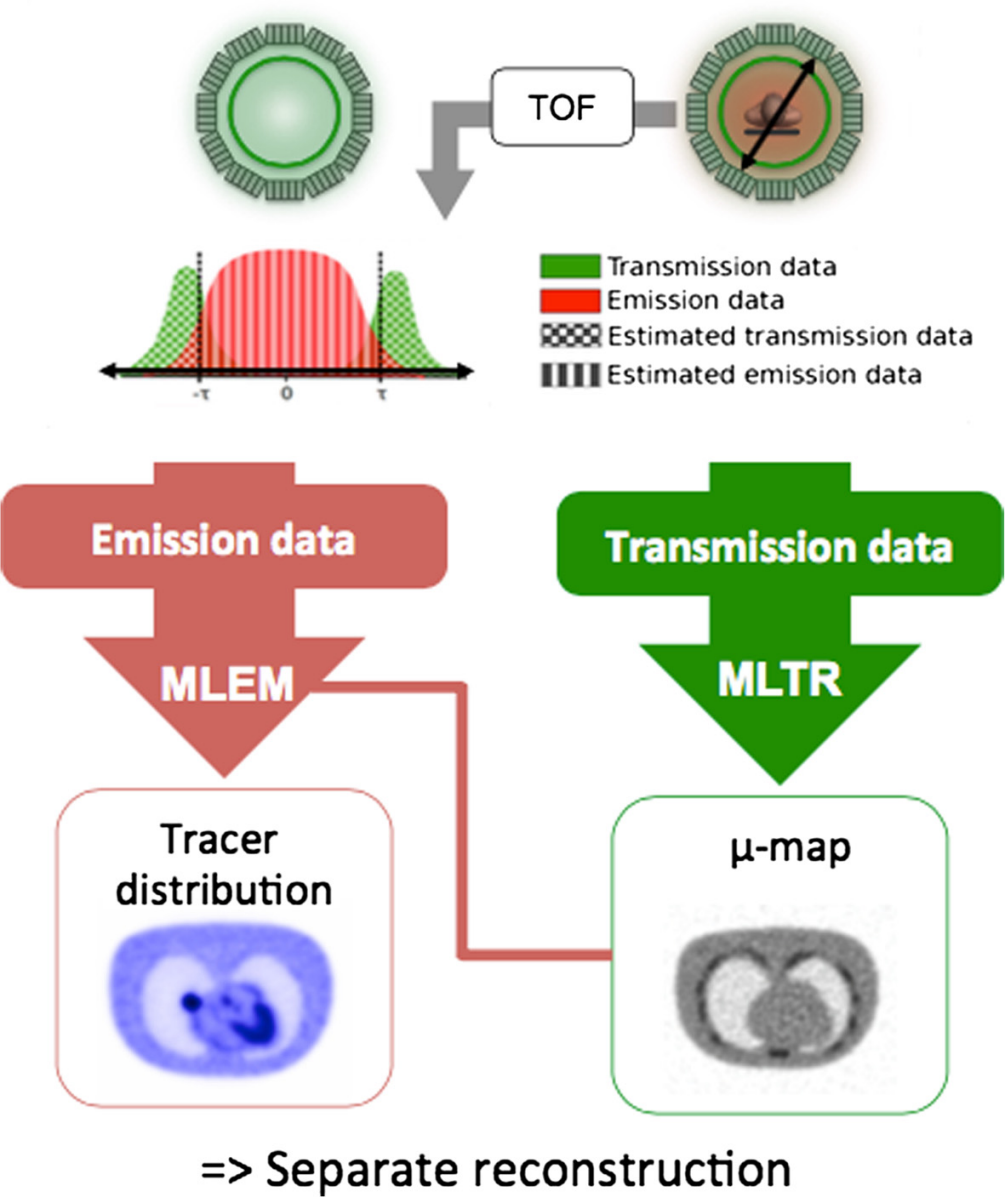
MLTR: the attenuation correction method based on the TOF separation of the transmission and the emission data

Due to the limitations associated with MR-based attenuation correction in PET/MR [101], the option of performing attenuation correction without the MR information is being investigated. As such, there is a renewed interest in external positron sources for transmission scanning. The TOF information makes it feasible to separate events based on their spatial origin and therefore to perform a simultaneous acquisition of transmission and emission events [102]. The transmission data, together with a blank scan of the source without object, can be reconstructed to obtain an attenuation coefficient distribution, for instance, by a maximum likelihood reconstruction of the transmission data (MLTR) [80] (Fig. 8). Compared to MR-based attenuation correction, the transmission-based attenuation correction has the additional advantages of not suffering from MR artifacts, MR truncation, segmentation errors, or the need for attenuation templates for MR coils and patient bed. The TOF resolution of current clinical PET systems (∼600 ps) however does not allow for complete separation of transmission and emission events, as was reported in [103]. The contamination of emission events in the transmission data may result in a non-uniform scaling in the reconstructed attenuation map. It is expected however that with future improvement of TOF resolution, this issue will become less prominent.

#### Low count studies

The majority of PET studies are done with FDG or other F-18-based molecules (choline, FLT). The positron decay fraction of F-18 is nearly 100 %. In general, these molecules result in relative high sensitivity studies, and TOF is used to reduce imaging time or improve the signal. For certain molecules, other isotopes are easier to use. Isotopes with very short half-lives like ^15^*O*, ^13^*N*, and ^82^*Rb* or short half-lives like ^11^*C* are often injected in high doses hitting the limits of PET electronics, leading to a high random fraction. Their short half-life however limits the acquisition time and the number of counts. Therefore, these can also benefit from the increased effective sensitivity of TOF-PET.

There is also a wide variety of isotopes for PET, which only have a small proportion of the decays leading to positron emission (called low branching ratio). This evidently reduces the effective sensitivity of PET. In some cases, they also have singles emitted close to 511 keV and spurious coincidences (singles associated with a positron decay). Two of these isotopes are I-124 (23 % branching ratio) and Y-86 (32 % branching ratio) and others are available. In general, these emission properties result in larger fraction of contaminating coincidences (combinations of singles from the positron annihilation with non-annihilation gammas). TOF will also for these isotopes result in a better SNR for the same number of counts. An additional benefit is the reduction of the number of effective randoms and scatters. Only random and scattered coincidences positioned by TOF inside or close to the object will contribute noise to the reconstructed image. Specific corrections are always needed for isotopes suffering from contamination. The TOF nature may require adapted corrections like I-124 [104]. An extreme example of these isotopes is Y-90 (the most common isotope used for radionuclide therapy). Due to a very small positron fraction (36 ×10^−4^ %), it is challenging to use it for PET imaging. In the case of radio-embolisation of liver tumors, the amount of administered tracer is high and very localized. First promising results for Y-90 TOF-PET have been presented recently in [105]. Another typical example of PET scan with extremelly low counts (< 10^5^ true events) is a dosimetry scan right after hadron therapy. Work [106], performed with a phantom, also shows that image quality in the case of therapy monitoring with an immediate PET scan can be improved by using TOF-PET and point-spread function modeling even at random fraction as high as > 95 %. More research is needed to determine the potential gain of TOF for the different isotopes.

#### Limited angle tomography

Image reconstruction is based on the use of projections from a range of angles, which is typically 0° to 180° or up to 360°. In general, PET systems are composed of complete ring detectors and data from all angles is available to obtain an accurate reconstruction. The problem of obtaining accurate reconstructions from a limited number of projections is more difficult to solve. The situation can appear in some scanners, where not a complete ring system can be used to collect data.

##### Dedicated breast imaging

**Fig. 9:**
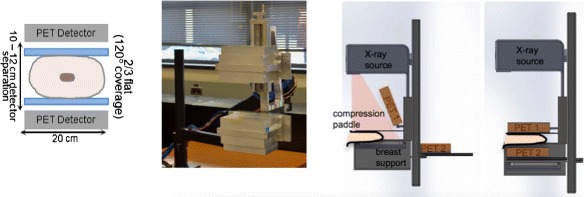
A limited angle design for a breast TOF-PET scanner. From left to right: the schematic of detectors (with compression paddles) and limited angle acquisition, physical detector modules in lab (evaluated with phantoms and illustration of integration of PET with X-ray used for tomosynthesis imaging

Different dedicated breast PET systems have been proposed during the last years. One of the reasons to use dedicated devices is the lower cost of these systems, their higher sensitivity, improved spatial resolution, and reduced attenuation. Designing a complete ring dedicated breast scanner is difficult and limited angle systems are preferable as they have more flexibility in placement around the patient. A partial (split) ring instrument also allows an integration of PET with either mammography or tomosynthesis to provide complementary imaging, see Fig. 9. This design is however inherently limited by longitudinal blurring in the direction orthogonal to the detector due to uncompleted data. The number of required angles to obtain the same image resolution is reduced, as better TOF information is available [25]. The availability of TOF information reduces also the influence of missing angles on the reconstructed image. The first simulated data of a breast scanner design based on TOF-PET [107] has shown the reduction of blurring by the incorporation of TOF information and was evaluated using a hot lesion phantom [108]. In [109], it was shown that 600 ps is appropriate for providing a tomographic image in a 2/3 angular coverage design. With 300 ps, similar precision was obtained as in a non-TOF scanner with full coverage.

##### In-beam PET

Another application of TOF-PET is in-beam PET [110] for hadron therapy.

It is a promising technology for determining the dose delivered to the target and to surrounding tissues. During the hadron therapy, patients are irradiated inside a PET scanner. Positron emitters are created by the (*p,n*) or (*p*,2*n*) interactions of the incoming protons with *C*^12^ and *O*^16^ inside the body. In the case of irradiation with carbon ions, positron emitters are created by fragmentation of the carbon projectile.

This is a typical situation where PET images have to be generated with a limited amount of counts and within a short time frame to allow feedback. The isotopes generated by this type of therapy are *C*^11^, *O*^15^, *C*^10^, and *N*^13^. These isotopes have short half-lives of 20 min, 2 min, 20 s, and 10 min making in-beam PET almost necessary. To integrate a PET into a treatment center, the system has to be a limited angle scanner. Normally, such systems result in poor spatial resolution in certain directions thus limiting the measured accuracy of the derived dose. TOF information helps to decrease these image degrading effects. Furthermore, the localization provided by TOF-PET also reduces the effect of outside FOV activity by reducing the amount of random.

Within the Envision project (http://envision.web.cern.ch/ENVISION/), two different technologies have been investigated for implementing TOF-PET for in-beam imaging. For crystal-based systems, cerium-doped lutetium-yttrium oxyorthosilicate (LYSO:Ce) was chosen as the best and cost-effective scintillation material. These were readout by SiPMs [111] and TOF resolutions of about 235 ps CRT FWHM for two teflon-wrapped LYSO:Ce crystals (15-mm thick) were obtained [112]. As an alternative low-cost detector technology, multigap RPC (MRPC) detectors were investigated [113]. These are composed of six thin glass plates acting as a mechanical separator for electrodes and, additionally, creating several gas gaps in between them. The gamma detection principle of RPC is the following. Firstly, 511-keV gamma photons interact with the electrodes producing energetic electrons. The electron might reach the gas gap and, in the case of success, initiates avalanche multiplication thus producing a signal. The bigger the number of the electrodes, the higher the detection efficiency of the RPC. Therefore, geometry of the MRPC allows to place many electrodes in a compact volume thus providing high detection efficiency for 511 keV gammas. Experimentally, TOF resolution of 560 ps CRT FWHM were obtained. These hardware studies were complemented by an extensive simulation study to investigate the limits of both technologies. The simulation [114] results indicate a superior performance of crystal-based detectors due to their higher stopping power, resulting in higher sensitivity. The results show that both systems can be used to detect 3-mm-big deviation in the expected location of Bragg peak in intended proton therapy dose. These deviations can arise due to errors in patient positioning or/and calculations of proton stopping power from CT-provided attenuation coefficients. They cause insufficient dose delivery to the tumor and extra dose expose of healthy tissues.

**Fig. 10:**
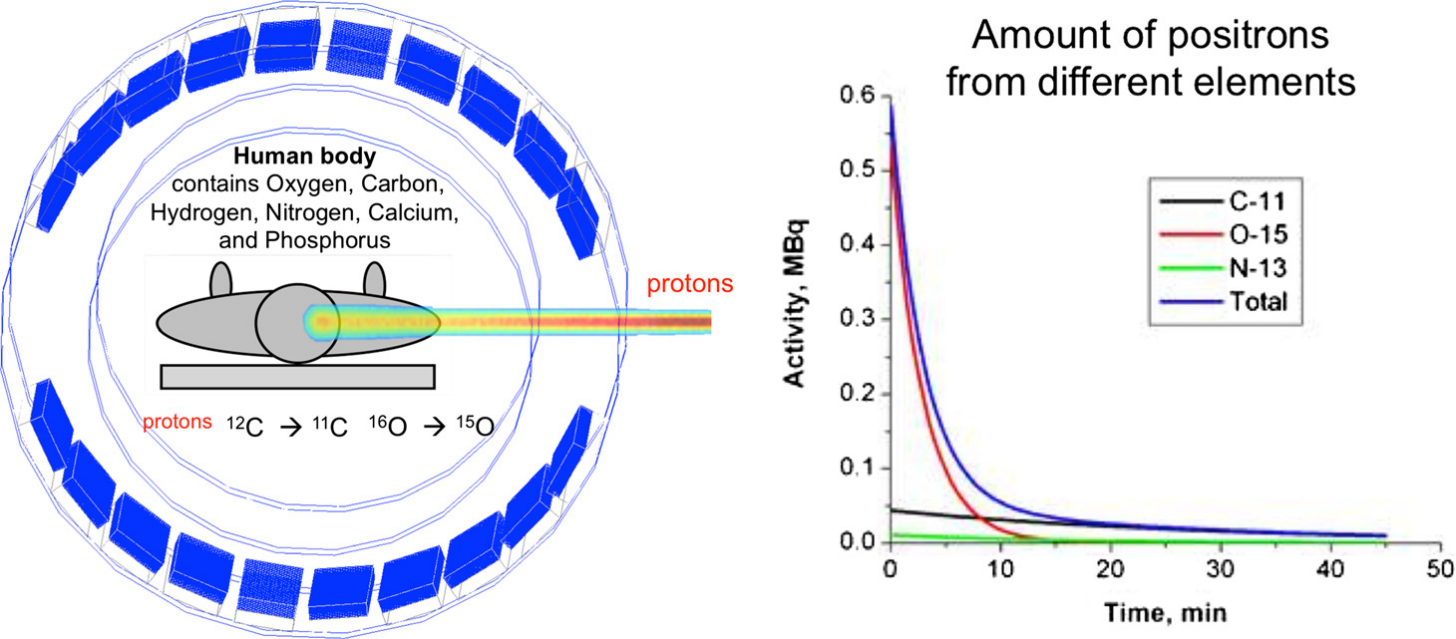
Open ring design of in-beam PET

In order to collect data directly during particle therapy, new designs of limited-angle in-beam TOF-PET system (Fig. 10), such as a PET scanner with an axial gap and a axially skewed complete ring, have been proposed. Both geometries allow the beam to pass through the patient without hitting the detectors of the PET scanner. Such systems have the advantage of generating complete data in the area of irradiation [115].

## Conclusions

TOF is now available on the high-end PET systems of the different major companies in medical imaging. The combination of fast scintillators, PMTs, and electronics has enabled these systems to reach timing resolutions in the range of 300–600 ps and to reliably perform in the clinic. New electronics (like waveform sampling), better PMTs, or solid-state detectors like SiPMs can even further improve the timing resolution. New prototypes, which aim to improve TOF resolution or/and build simultaneous PET/MR hybrids, are also under development.

Calibration and TOF reconstruction techniques have been improved in the last 10 years after the introduction of the first commercial TOF-PET scanners. There is still room for improvement by better kernel modeling and offset corrections. Different studies have characterized TOF gain using simulated or measured phantoms and evaluations on patient studies have also been performed, to assess how the gain translates to a clinical benefit. The large availability of these systems will enable to collect more patient studies, extend the evaluation towards larger groups, and determine the potential clinical impact on health outcome. New applications for PET, such as dedicated breast imaging, PET with isotopes which have low branching ratio and in-beam PET, should benefit from better image quality, which is achieved by making use of the higher effective sensitivity, the reduced influence of scattered and random events, and the reduced effects of limited angle data.
